# Cancer Stemness-Based Prognostic Immune-Related Gene Signatures in Lung Adenocarcinoma and Lung Squamous Cell Carcinoma

**DOI:** 10.3389/fendo.2021.755805

**Published:** 2021-10-21

**Authors:** Na Li, Yalin Li, Peixian Zheng, Xianquan Zhan

**Affiliations:** ^1^ Department of Radiation Oncology, and Shandong Key Laboratory of Radiation Oncology, Shandong Cancer Hospital and Institute, Shandong First Medical University, Jinan, China; ^2^ Medical Science and Technology Innovation Center, Shandong First Medical University, Jinan, China; ^3^ Gastroenterology Research Institute and Clinical Center, Shandong First Medical University, Jinan, China

**Keywords:** lung cancer, cancer stemness, tumor microenvironment, immune-related gene signature, clinical characteristics

## Abstract

**Background:**

Cancer stem cells (CSCs) refer to cells with self-renewal capability in tumors. CSCs play important roles in proliferation, metastasis, recurrence, and tumor heterogeneity. This study aimed to identify immune-related gene-prognostic models based on stemness index (mRNAsi) in lung adenocarcinoma (LUAD) and lung squamous cell carcinoma (LUSC), respectively.

**Methods:**

X-tile software was used to determine the best cutoff value of survival data in LUAD and LUSC based on mRNAsi. Tumor purity and the scores of infiltrating stromal and immune cells in lung cancer tissues were predicted with ESTIMATE R package. Differentially expressed immune-related genes (DEIRGs) between higher- and lower-mRNAsi subtypes were used to construct prognostic models.

**Results:**

mRNAsi was negatively associated with StromalScore, ImmuneScore, and ESTIMATEScore, and was positively associated with tumor purity. LUAD and LUSC samples were divided into higher- and lower-mRNAsi groups with X-title software. The distribution of immune cells was significantly different between higher- and lower-mRNAsi groups in LUAD and LUSC. DEIRGs between those two groups in LUAD and LUSC were enriched in multiple cancer- or immune-related pathways. The network between transcriptional factors (TFs) and DEIRGs revealed potential mechanisms of DEIRGs in LUAD and LUSC. The eight-gene-signature prognostic model (ANGPTL5, CD1B, CD1E, CNTFR, CTSG, EDN3, IL12B, and IL2)-based high- and low-risk groups were significantly related to overall survival (OS), tumor microenvironment (TME) immune cells, and clinical characteristics in LUAD. The five-gene-signature prognostic model (CCL1, KLRC3, KLRC4, CCL23, and KLRC1)-based high- and low-risk groups were significantly related to OS, TME immune cells, and clinical characteristics in LUSC. These two prognostic models were tested as good ones with principal components analysis (PCA) and univariate and multivariate analyses. Tumor T stage, pathological stage, or metastasis status were significantly correlated with DEIRGs contained in prognostic models of LUAD and LUSC.

**Conclusion:**

Cancer stemness was not only an important biological process in cancer progression but also might affect TME immune cell infiltration in LUAD and LUSC. The mRNAsi-related immune genes could be potential biomarkers of LUAD and LUSC. Evaluation of integrative characterization of multiple immune-related genes and pathways could help to understand the association between cancer stemness and tumor microenvironment in lung cancer.

## Introduction

Non-small cell lung cancer (NSCLC) mainly included lung adenocarcinoma (LUAD, 40%), lung squamous cell carcinoma (LUSC, 30%), and large cell carcinoma (15%) (1). LUAD and LUSC had different characteristics in the following aspects, including mutation models, pathogenesis, molecular mechanisms, treatment plans, and personalized medical services (2). Cancer stem cells (CSCs) were a population of cells in tumors with self-renewal and infinite proliferation capability and could be the resource to generate heterogeneous tumor cells (3). CSCs played an important role in tumor survival, proliferation, epithelial-to-mesenchymal transition, invasiveness and migration capability, metastasis, recurrence, and therapy resistance (4). According to the theory of CSCs, CSCs had the ability to escape from antitumor therapies; thus, CSCs could not be killed by conventional chemotherapies, which only kill the bulk of differentiated and differentiating cancer cells (5). In this process, CSCs might express a variety of resistant molecules and mediate some resistance-related pathways, including polycomb group transcriptional repressor Bmi-1 pathway, Notch pathway, sonic hedgehog, Wnt pathway, phosphoinositide 3-kinase (PI3K)/AKT, tyrosine kinase receptors pathways [such as epidermal growth factor receptor (EGFR), fibroblast growth factor receptor (FGFR), hepatocyte growth factor receptor (HGFR)/MET, insulin-like growth factor receptor (IGFR), and platelet-derived growth factor (PDGF)], and transforming growth factor β (TGFβ)/SMAD pathway (6). CSCs were commonly identified with fluorescence-activated cell sorting plus specific antibodies binding to CSC-surface markers (ALDH1A1, CD24, CD34, CD44, CD133, and EPCAM) (7, 8). CSCs were found in multiple cancers, including lung cancer, breast cancer, colorectal cancer, liver cancer, prostate cancer, and blood tumor (9). Recent studies found that lung resident epithelia such as variant club cells, basal cells, club cells, and alveolar epithelial type-2 cells had facultative stem cell reparative activities (10). Additionally, the CSC theory introduced a new concept—mRNA expression-based stemness index (mRNAsi) based on machine learning algorithm (11). The mRNAsi was calculated with the transcriptomic expression profile of tumor samples from The Cancer Genome Atlas (TCGA) according to transcriptomic datasets of non-transformed pluripotent stem cells and their differentiated progeny (12). CSC theory proposed that tumor tissue contained its own cancer itself. Therefore, CSC theory might cast new lights on the nature of tumors and clinical differentiation therapy between LUAD and LUSC, respectively.

The development of tumor immunotherapy promoted the applications of immune checkpoint inhibitors in NSCLC, such as anti-PD-1/PD-L1 immune checkpoint therapy (13). Currently, eight kinds of PD-1/PD-L1 checkpoint inhibitors were approved worldwide, including six kinds of PD-1 checkpoint inhibitors [Merck Keytruda, Bristol-Myers Squibb Opdivo, Pfizer/Merck Bavencio, Sanofi/regenerative Libtayo, Jun Tolpalimab (Treplimab), and Cinnabi/Lilidabo Shu^®^ (Tyvyt, Sintelimab)] and two kinds of PD-L1 checkpoint inhibitors (Roche Tecentriq and AstraZeneca Imfinzi) (14). With the renaissance of anticancer immunotherapy and the occurrence of CSC escape from antitumor therapies, it might be essential to further explore the interplay between tumor immune microenvironment (TME) and CSCs to discover novel cancer approaches to treat drug resistance and immune escape of CSCs (15). Recent studies found that mRNAsi was closely related to TME and that higher mRNAsi was identified in many tumors with a lower-expressed PD-L1 and a reduced leukocyte fraction (11). Immunotherapy strategy largely depended on the infiltrated immune cells and upregulated PD-L1. However, those cancers with higher mRNAsi might be less susceptible to immune checkpoint blockade therapies, which suggested a potential mechanism of immune evasion (16). TME contained various cell types such as monocytes, natural killer (NK) cells, macrophages, eosinophils, dendritic and mast cells, and neutrophils (17). The relationships between these cell types and CSCs were assessed in tumors; for example, stem-cell markers (Nanog, Lgr5, CD44v6, and ALDH1A1) were highly associated with immune cell counts, which revealed that that cancer stemness and immune state should be considered as a whole (18). Additionally, some cytokine signals were also important for CSCs; for example, IL33 could mediate stem cell genes to stimulate cell sphere formation and increase tumor resistance to chemoradiotherapy, and IL33 activated OCT3/4, NOTCH3, and NANOG and enhanced transcription factor (TF) c-Jun that binds to the promoters of hub stem-cell-related genes (19). Moreover, CSCs had immunomodulatory capabilities and communicated with TME through producing immune system inhibitory factors. Thus, CSCs could interact with immune checkpoint molecules, such as Tim3, CD47, CTLA4, PDL-1, and LAG3, to protect cancer cells from immune clearance (20). Therefore, it was necessary to study the correlation between stemness (mRNAsi) and immunity (the fraction of immune cell populations, activation state of immune cells, or alterations of immune-related genes and pathways).

**Figure 1:**
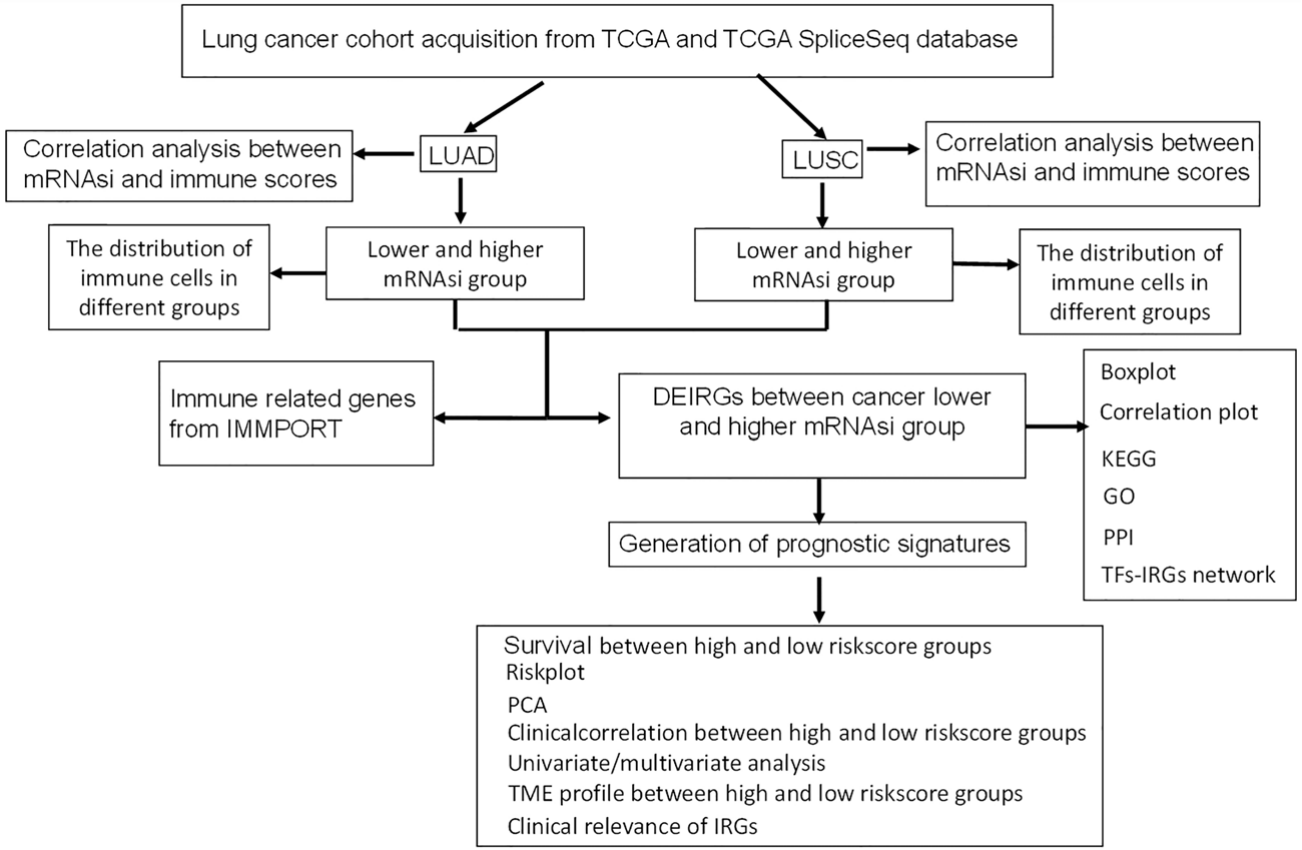
Flow chart for identification of mRNAsi-related immune signatures in LUAD and LUSC.

This study aimed to identify prognostic immune-related gene model related to stem cell characteristics based on mRNAsi in LUAD and LUSC, respectively. The immune cell types were profiled with CIBERSORT in lung cancers, which provided insights into the relationships between mRNAsi and infiltrating immune system cells and between mRNAsi and immune-related genes. Moreover, the constructed network between TFs and stemness-related differently expressed immune-related genes (DEIRGs) revealed the potential mechanisms of stemness-related DEIRGs in LUAD and LUSC, respectively. The overall experimental flow chart was showed for this study (**Figure 1**), and this method would also be easy to transit for other cancer studies.

## Materials and Methods

### Data Processing

The TCGA RNA-seq data were obtained from tissue of origin from solid biopsy of lung cancer patients. Level 3 RNA-seq data and corresponding clinical characteristics were downloaded from TCGA website (https://portal.gdc.cancer.gov/). The gene expression missing value (expression = 0) more than 20% was excluded with the pretreatment. The immune-related genes (IRGs) were selected from ImmPort database (https://www.immport.org/shared/home). The corresponding mRNAsi values were downloaded from supplementary materials of one published article (https://www.ncbi.nlm.nih.gov/pmc/articles/PMC5902191/); more specifically, the study used an innovative one-class logistic regression machine learning algorithm (OCLR) to extract transcriptomic and epigenetic feature sets that were derived from non-transformed pluripotent stem cells and their differentiated progeny. The OCLR machine-learning algorithm was used to calculate mRNAsi values. The mRNAsi range was from 0 to 1, and the closer mRNAsi was to 1, the stronger was the characteristics of stem cells. Any sample that was missing mRNAsi was excluded. The mRNAsi, overall survival data, and expressions of immune-related genes in LUAD and LUSC are listed in **Supplementary Tables S1**, **S2**.

The X-tile software (version 3.6.1) was used to determine the best cutoff value of mRNAsi in the survival data from LUAD and LUSC, respectively (21). The principle of X-tile software was to group different mRNAsi values into truncation values for statistical analysis, and the result with the smallest p-value (p < 0.05) was considered as the best truncation value to divide mRNAsi into two groups (higher and lower mRNAsi groups); in other words, X-title software divided samples into higher and lower mRNAsi groups.

### Proportions of Immune Cells in LUAD and LUSC Based on CIBERSORT Method

To quantify the relative percentage of immune cells in lung cancer samples, LM22 gene signature and CIBERSORT algorithm were used for highly sensitive and specific discrimination of 22 human immune cell phenotypes. Gene expression profiles were prepared with standard annotation files, and data were uploaded to CIBERSORT web portal (http://cibersort.stanford.edu/), with the algorithm based on the LM22 signature and 1,000 permutations (**Supplementary Tables S3**, **S4**).

### Estimation of Tumor Purity and Infiltrating Cells in LUAD and LUSC

Tumor purity and the presence of infiltrating stromal and immune cells in tumor tissues were predicted with ESTIMATE R package that estimated stromal and immune cells in malignant tumor tissues based on gene expression data (**Supplementary Tables S5**, **S6**), which generated three scores, including (i) ImmuneScore representing the infiltration of immune cells in tumor tissue, (ii) StromalScore capturing the presence of stroma in tumor tissue, and (iii) ESTIMATEScore inferring tumor purity. Pearson correlation coefficient was used to test the correlation levels between these scores and mRNAsi. The distribution of immune cells was analyzed between higher and lower mRNAsi groups in LUAD and LUSC, respectively.

### Identification of DEIRGs Between Higher and Lower mRNAsi Groups

The limma package (http://bioinf.wehi.edu.au/limma/) was used for DEIRG analysis, and p < 0.05, false discovery rate (FDR) filter <0.05, and log (fold change) filter >0.58 were set as the threshold to select DEIRGs in LUAD or LUSC between higher and lower mRNAsi groups (**Supplementary Tables S7**, **S8**). The correlation levels between DEIRGs were analyzed with Corrplot R package (https://www.rdocumentation.org/packages/corrplot/versions/0.2-0/topics/corrplot).

### Functional and Pathway Enrichment Analyses of DEIRGs

All DEIRGs were analyzed with Kyoto Encyclopedia of Genes and Genomes (KEGG) pathway and Gene Ontology (GO) enrichment analyses, including biological processes (BPs), molecular functions (MFs), and cellular components (CCs). The clusterProfiler R package (http://bioconductor.org/packages/release/bioc/html/clusterProfiler.html) was used for gene-annotation enrichment analysis of DEIRGs between higher and lower mRNAsi groups, including statistically significant GO terms (**Supplementary Tables S9**, **S10**) and KEGG pathways (**Supplementary Tables S11**, **S12**), with p < 0.05 and false discovery rate (FDR) <0.05. The p-value of enrichment analysis was calculated based on 10,000 permutations, and FDR value was calculated with Benjamini–Hochberg multiple testing correction procedures. All DEIRGs were mapped in protein–protein interaction (PPI) network in the STRING database (https://string-db.org/) (**Supplementary Tables S13**, **S14**).

### Construction of TF-DEIRG Networks

TFs or sequence-specific DNA-binding factors were a cluster of proteins that could control the rate of transcription from DNA to mRNA, which was obtained from Cistrome Cancer database (http://cistrome.org/db/). TF gene expressions from TCGA database were matched with Cistrome Cancer database (**Supplementary Tables S15**, **S16**). TF-DEIRG network was visually presented with Cytoscape software based on correlation coefficient filter >0.4 and p-value filter < 0.05 (**Supplementary Tables S17**, **S18**).

### Lasso Regression Construction and Verification for LUAD

Cox proportional hazard regression model was used for overall survival (OS) analysis to evaluate impact of continuous variables (DEIRGs) on survival. Cox proportional hazard regression was performed with survival R package (https://www.rdocumentation.org/packages/survival/versions/3.2-3) to select OS-related DEIRGs with p<0.05 (**Supplementary Table 19**). Furthermore, OS-related DEIRGs were used for least absolute shrinkage and selection operator (lasso) regression. Lasso regression was a regression method that performed both variable selection and regularization to enhance the prediction accuracy and interpretability of the statistical model it produced. The best subset selection and the connections between lasso coefficient estimates were identified to construct the prognostic model. Lung cancer samples were divided into two groups (**Supplementary Table S20**) according to the median value of risk scores (high- and low-risk score groups). The Kaplan–Meier method was used to evaluate the availability of prognostic model between high- and low-risk score groups. Principal component analysis (PCA) was performed to measure classifications with risk sore. The distribution of immune cells was analyzed between high- and low-risk score groups in LUAD patients. The clinical data were obtained from TCGA database, including gender (male and female), age (aged ≤65 and >65), anatomic subdivision (R-lower, R-middle, R-upper, L-lower, L-middle, and L-upper), follow-up outcome (partial and complete remissions/responses, progressive and stable diseases), number pack years smoked (packs from 0.15 to 240), pathological T (tumor size, including T1, T2, T3,T4, and TX), pathological M (tumor metastasis, including M0, M1, and MX), pathological N (tumor lymph node metastasis, including N0, N1, N2, and NX), pathological stage (stages I, II, III, and IV), cancer status (tumor or tumor free), radiation therapy (no or yes), targeted molecular therapy (yes or no), and status (alive or dead) (**Supplementary Table S21**). Clinic correlation between high- and low-risk score groups was analyzed with pheatmap R package (http://bioconductor.org/packages/3.8/bioc/html/heatmaps.html). In addition, clinical characteristics (including gender, age at initial diagnosis, follow-up, anatomic subdivision, number pack years smoked, pathological stage, pathological T, pathological N, pathological M, cancer status, radiation therapy, and targeted molecular therapy) associated with OS were analyzed in LUAD patients with univariate and multivariate Cox regression models.

### Multivariate Cox Regression Analysis of LUSC

Multivariate Cox regression analysis was performed to calculate the regression coefficient for each DEIRG with statistical significance p < 0.05 to construct prognostic model, with survival R package (http://bioconductor.org/packages/devel/bioc/vignettes/survtype/inst/doc/survtype.html) to select OS-related DEIRGs (**Supplementary Table S22**). LUSC samples were divided into high- and low-risk score groups (**Supplementary Table S23**) according to the median value of risk scores. The Kaplan–Meier method was used to evaluate the availability of prognostic model between high- and low-risk score groups. PCA was performed to measure classifications with risk sore. The distribution of immune cells was analyzed between high- and low-risk score groups in LUSC patients. Clinic correlation between high- and low-risk score groups was analyzed with pheatmap R package. In addition, clinical characteristics (including age at initial diagnosis, anatomic subdivision, follow-up, gender, number pack years smoked, pathological M, pathological N, pathological T, pathological stage, cancer status, radiation therapy, and targeted molecular therapy) associated with OS (**Supplementary Table S24**) were analyzed with univariate and multivariate Cox regression models in LUSC patients.

### Correlation Between Gene Expressions in Prognostic Model and Clinical Characteristics

The correlation between gene expressions in prognostic model and clinical characteristics was performed with Beeswarm R package (https://www.rdocumentation.org/packages/beeswarm/versions/0.2.3/topics/beeswarm).The scatter plot showed DEIRG expressions for the TNM degree (pathological T, pathological N, and pathological M) and pathological stages of LUAD and LUSC cases, respectively (p < 0.05).

### Statistical Analysis

For between-group comparison, each p-value was calculated with unpaired Student *t*-test for normally distributed variables, and with Mann–Whitney U-test (namely, the Wilcoxon rank-sum test) for non-normally distributed variables, and statistical significance was set as p < 0.05. FDR and Benjamini–Hochberg for multiple testing was used for DEIRG, GO, and KEGG analyses. The Kaplan–Meier method was used to generate survival curves, and the log-rank (Mantel-Cox) test was used to evaluate statistical significance (p < 0.05). The hazard ratio was calculated for univariate and/or multivariate Cox proportional hazard regression models (p < 0.05).

## Results

### High and Low mRNAsi Subtypes in LUAD and LUSC

**Figure 2:**
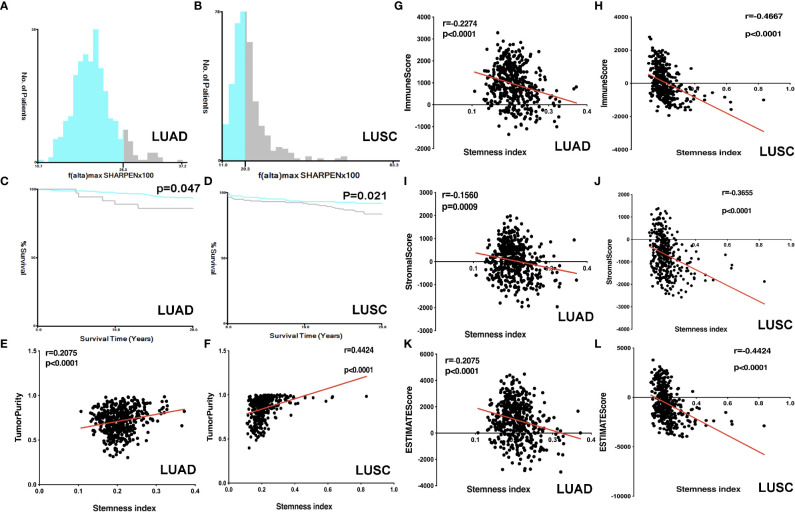
Correlation analysis between ESTIMATE scores and mRNAsi. **(A)** The samples were divided into two groups with X-tile in LUAD (x = mRNAsi × 100 and y = number of patients). **(B)** The samples were divided into two groups with X-tile in LUSC (x = mRNAsi × 100 and y = number of patients). **(C)** Kaplan–Meier survival curves show that the low-mRNAsi group had a better OS rate than the high mRNAsi group in LUAD. **(D)** Kaplan–Meier survival curves show that the low mRNAsi group had a better OS rate than the high mRNAsi group in LUSC. **(E)** The correlation between tumor purity and mRNAsi in LUAD. **(F)** The correlation between tumor purity and mRNAsi in LUSC. **(G)** The correlation between immunescore and mRNAsi in LUAD. **(H)** The correlation between immunescore and mRNAsi in LUSC. **(I)** The correlation between stromalscore and mRNAsi in LUAD. **(J)** The correlation between stromalscore and mRNAsi in LUSC. **(K)** The correlation between ESTIMATEscore and mRNAsi in LUAD. **(L)** The correlation between ESTIMATEscore and mRNAsi in LUSC.

**Figure 3:**
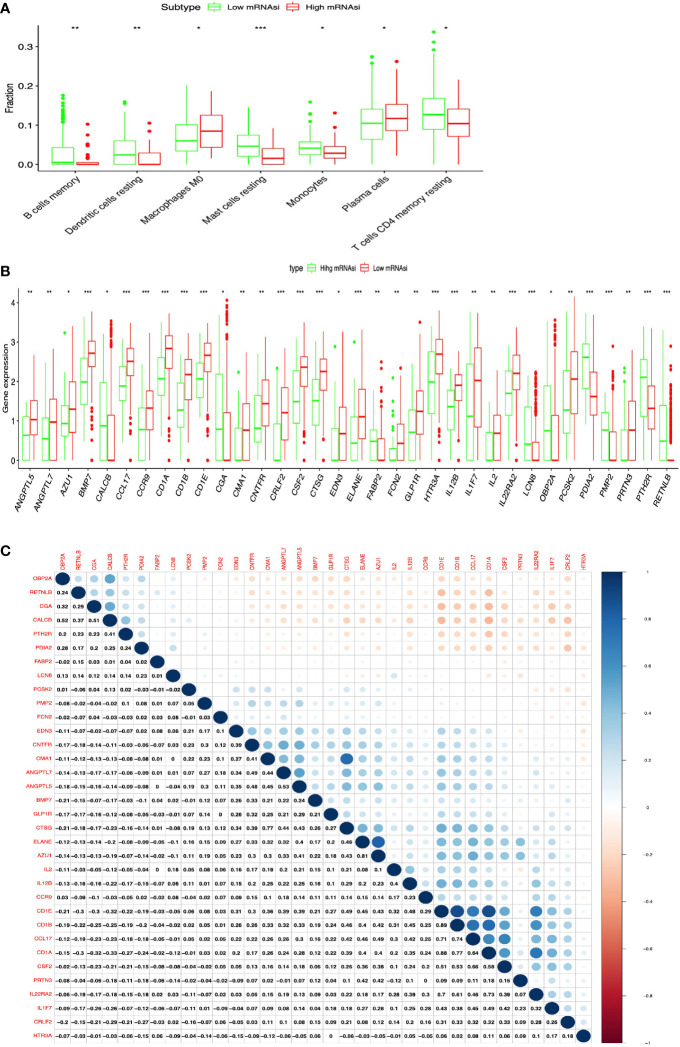
Identification of DEIRGs between higher- and lower-mRNAsi groups in LUAD. **(A)** The distribution of immune cells between higher- and lower-mRNAsi groups. **(B)** DEIRGs between higher- and lower-mRNAsi groups. **(C)** The correlation between DEIRGs. *p < 0.05, **p < 0.01, ***p < 0.001.

**Figure 4:**
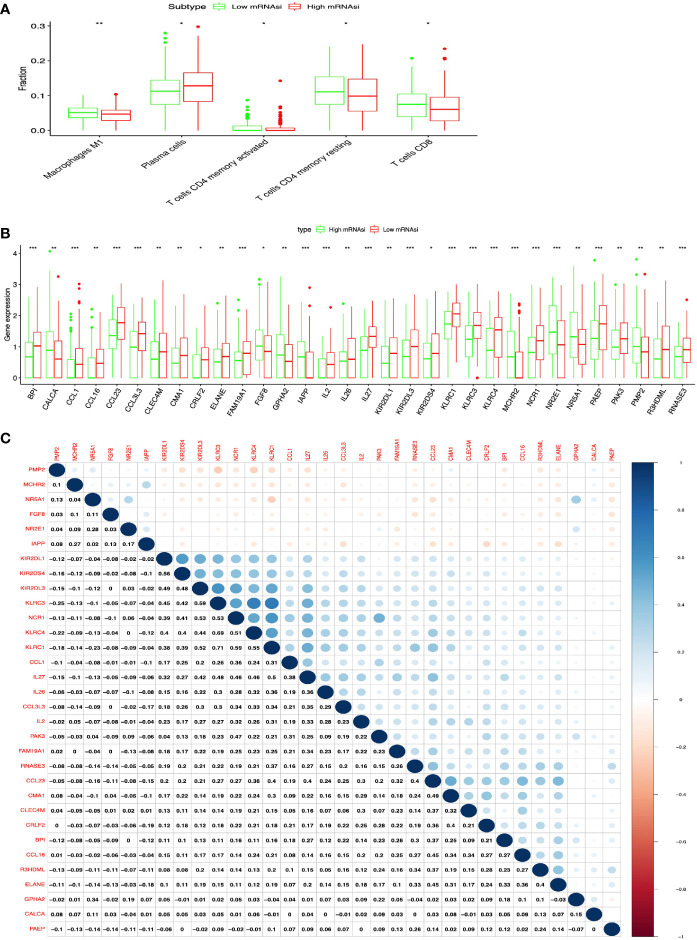
Identification of DEIRGs between higher- and lower-mRNAsi groups in LUSC. **(A)** The distribution of immune cells between higher- and lower-mRNAsi groups. **(B)** DEIRGs between higher- and lower-mRNAsi groups. **(C)** The correlation between DEIRGs. *p < 0.05, **p < 0.01, ***p < 0.001.

The lung cancer survival data and corresponding mRNAsi data were from LUAD (n=452) and LUSC (n = 363) patients. X-tile software was used to divide samples into high- and low-mRNAsi subtypes, with cutoff value = 0.263 (χ^2^ = 3.0935) in LUADs (**Figure 2A**) and cutoff value = 0.203 (χ^2^ = 5.3207) in LUSCs (**Figure 2B**), respectively (LUAD: n = 39 for high mRNAsi group and n = 413 for low mRNAsi group; LUSC: n = 173 for high mRNAsi group and n = 190 for low mRNAsi group) (**Supplementary Tables S1**, **S2**). The patients with high mRNAsi showed significant poor prognosis in LUAD (p = 0.047) and LUSC (p = 0.021) (**Figures 2C, D** **)**. The mRNAsi range was from 0 to 1, and the closer it was to 1, the stronger the characteristics of stem cells were. The abundance of each TME cell infiltration based on RNA expression data was calculated in LUAD and LUSC with CIBERSORT, respectively (**Supplementary Tables S3**, **S4**), including naïve B cells, B cells memory, plasma cells, T cells CD8, T cells CD4 memory resting, T cells CD4 memory activated, T cells follicular helper, Tregs, NK cells resting, NK cells activated, monocytes, macrophages M0, macrophages M1, macrophages M2, dendritic cells resting, dendritic cells activated, mast cells resting, mast cells activated, eosinophils, and neutrophils. ESTIMATE R package estimated stromal and immune cells in LUAD and LUSC tissues with ImmuneScore, StromalScore, ESTIMATEScore, and tumor purity (**Supplementary Tables S5**, **S6**). The positive associations were found between mRNAsi and tumor purity (**Figures 2E, F** **)**. The negative associations were found between mRNAsi and ImmuneScore (**Figures 2G, H** **)**, between mRNAsi and StromalScore (**Figures 2I, J** **)**, and between mRNAsi and ESTIMATEScore (**Figures 2K, L** **)**. Additionally, the distribution of immune cells between high- and low-mRNAsi subtypes were significantly different in LUAD, including B cells memory (p = 0.002), dendritic cells resting (p = 0.005), macrophages M0 (p = 0.025), mast cells resting (p = 0.0008), monocytes (p = 0.039), plasma cells (p = 0.014), and T cells CD4 memory resting (p = 0.034) (**Figure 3A**). Among them, the distribution of B cells memory, dendritic cells resting, mast cells resting, monocytes, and T cells CD4 memory resting were significantly higher in low-mRNAsi subtype (p < 0.05), and the distribution of macrophages M0 and plasma cells were significantly higher in high-mRNAsi subtype (p < 0.05) (**Figure 3A**). The distribution of immune cells between high- and low-mRNAsi subtypes were significantly different in LUSC, including macrophages M1 (p = 0.003), plasma cells (p = 0.014), T cells CD4 memory resting (p = 0.023), T cells CD4 memory activated (p = 0.038), and T cells CD8 (p = 0.035) (**Figure 4A**). Among them, the distribution of macrophages M1, T cells CD4 memory resting, T cells CD4 memory activated, and T cells CD8 were significantly higher in low-mRNAsi subtype (p < 0.05), and the distribution of plasma cells was significantly higher in high-mRNAsi subtype (p < 0.05) (**Figure 4A**).

### DEIRGs Between High- and Low-mRNAsi Subtypes in LUAD and LUSC

In total, 34 DEIRGs were identified between high- and low-mRNAsi subtypes in LUAD (**Figure 3B** and **Supplementary Table S7**), including 9 upregulated DEIRGs (OBP2A, CALCB, PTH2R, PDIA2, FABP2, CGA, PMP2, LCN8, and RETNLB) and 25 downregulated DEIRGs (CMA1, CRLF2, ELANE, IL2, GLP1R, PRTN3, EDN3, CD1B, ANGPTL5, FCN2, IL12B, CTSG, CCR9, PCSK2, CSF2, CD1A, ANGPTL7, AZU1, CCL17, CNTFR, CD1E, IL22RA2, HTR3A, IL1F7, and BMP7). In addition, high correlations (correlation coefficient > 0.4, and p < 0.05) occurred between these DEIRGs in LUAD, for example, CALCB and OBPIA (Cor = 0.52, p = 0.004), CALCB and CGA (Cor = 0.51, p = 0.034), CMA1 and CNTFR (Cor = 0.41, p = 0.028), ANGPTL7 and CMA1 (Cor = 0.49, p = 0.003), ANGPTL7 and CNTFR (Cor = 0.44, p = 0.021), ANGPTL5 and CMA1 (Cor = 0.48, p = 0.014), ANGPTL5 and CNTFR (Cor = 0.45, p = 0.032), ANGPTL7 and ANGPTL5 (Cor = 0.53, p = 0.001), ELANE and CTSG (Cor = 0.46, p = 0.014), AZU1 and CTSG (Cor = 0.43, p = 0.037), CD1A and CCL17 (Cor = 0.40, p = 0.008), and CD1E and IL12B (Cor = 0.48, p = 0.017) (**Figure 3C**).

In total, 32 DEIRGs were identified between high- and low-mRNAsi subtypes in LUSC (**Figure 4B** and **Supplementary Table S8**), including 8 upregulated DEIRGs (PMP2, NR2E1, IAPP, CALCA, FGF8, GPHA2, NR5A1, and MCHR2) and 24 downregulated DEIRGs (CCL1, BPI, IL2, IL27, CCL16, KLRC4, IL26, NCR1, FAM19A1, KLRC3, KIR2DS4, PAEP, RNASE3, R3HDML, CRLF2, CCL3L3, ELANE, KIR2DL1, KLRC1, CMA1, CLEC4M, CCL23, KIR2DL3, and PAK3). In addition, high correlations (correlation coefficient > 0.4 and p < 0.05) occurred between these DEIRGs in LUSC, for example, KIR2DS4 and KIRIPL1 (Cor = 0.56, p = 0.013), KLRC3 and KIRIDL3 (Cor = 0.45, p = 0.024), KLRC3 and KIRIDL1 (Cor = 0.42, p = 0.038), KLRC3 and KIRIDS4 (Cor = 0.59, p = 0.001), CMA1 and CCL23 (Cor = 0.49, p = 0.044), CRLF2 and CMA1 (Cor = 0.40, p = 0.014), ELANE and R3HDML (Cor = 0.40, p = 0.037), and KIRC1 and NCR1 (Cor = 0.59, p = 0.004) (**Figure 4C**).

### DEIRG-Mediated Signaling Pathways and Their Functional Characteristics in LUAD and LUSC

**Figure 5:**
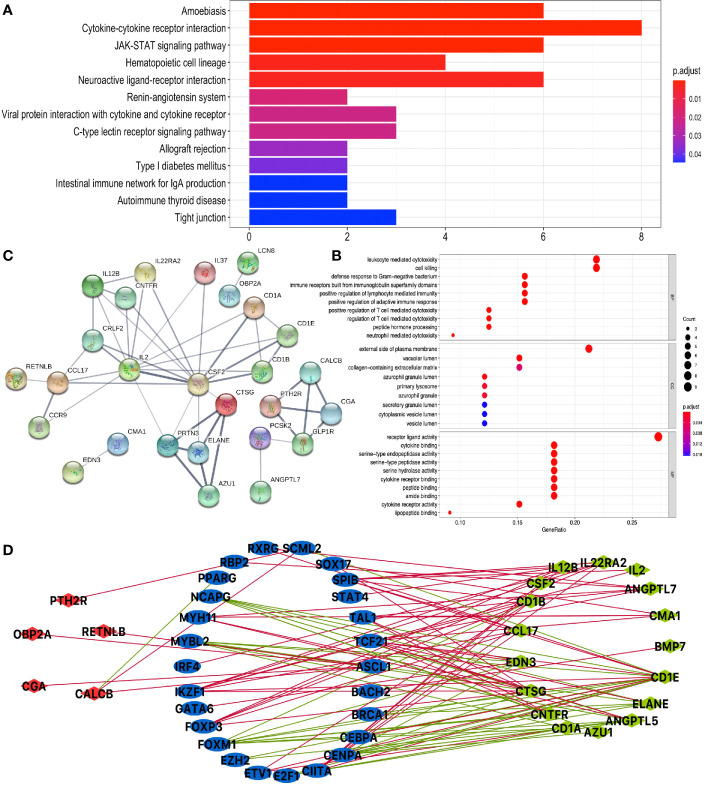
Function enrichment analysis of DEIRGs and TF-DEIRG network in LUAD. **(A)** KEGG pathway analysis of DEIRGs. **(B)** GO analysis of DEIRGs. The bar plots and bubble plots show the top terms of biological processes (BPs), cellular components (CCs), and molecular functions (MFs). **(C)** PPI network of DEIRGs. **(D)** TF-DEIRG network in LUAD. The blue circle refers to the TFs, the red circle refers to the upregulated DEIRGs, and the green circle refers to the downregulated DEIRGs. The red line is the positive correlation between TFs and DEIRGs. The green line is the negative correlation between TFs and DEIRGs.

KEGG pathway analysis of DEIRGs in LUAD identified 13 significant pathways, including cytokine–cytokine receptor interaction, JAK-STAT signaling pathway, hematopoietic cell lineage, neuroactive ligand–receptor interaction, renin–angiotensin system, viral protein interaction with cytokine and cytokine receptor, C-type lectin receptor signaling pathway, allograft rejection, type I diabetes mellitus, intestinal immune network for IgA production, autoimmune thyroid disease, and tight junction (**Figure 5A** and **Supplementary Table S9**). GO analysis of DEIRGs in LUAD identified 142 significant BPs, 9 CCs, and 28 MFs (**Figure 5B** and **Supplementary Table S11**). Protein–protein interaction (PPI) network showed some of the high combined scores (combined score >0.9) between those DEIRGs in LUAD; for example, CSF2 and IL2, ELANE and CTSG, ELANE and AZU1, PRTN3 and AZU1, ELANE and PRTN3, AZU1 and CTSG, PRTN3 and CTSG, GLP1R and PTH2R, CGA and PTH2R, CGA and GLP1R, CALCB and GLP1R, CALCB and PTH2R, and CGA and CALCB (**Figure 5C** and **Supplementary Table S13**).

**Figure 6:**
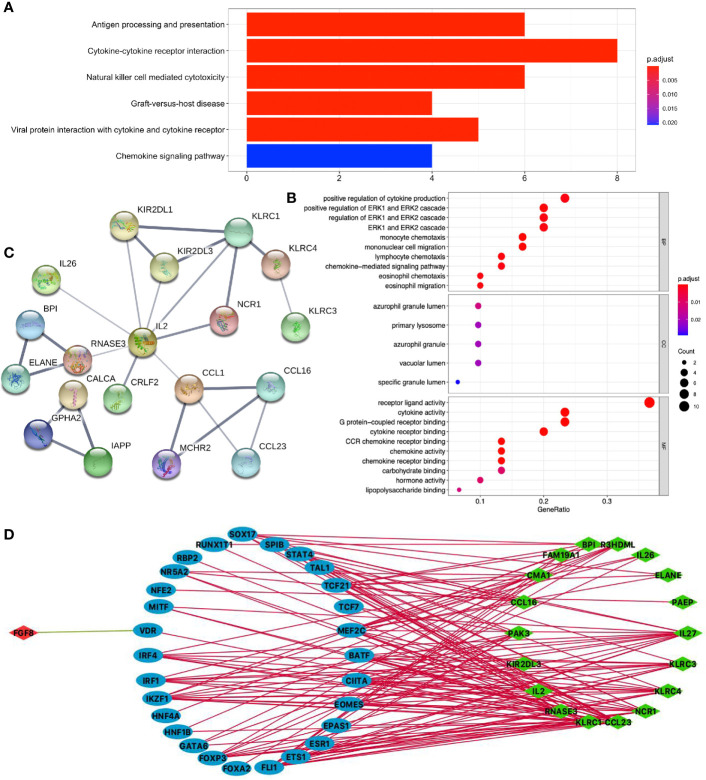
Function enrichment analysis of DEIRGs and TF-DEIRG network in LUSC. **(A)** KEGG pathway analysis of DEIRGs. **(B)** GO analysis of DEIRGs. The bar plots and bubble plots show the top terms of biological processes (BPs), cellular components (CCs), and molecular functions (MFs). **(C)** PPI network of DEIRGs. **(D)** TF-DEIRG network in LUSC. The blue circle refers to the TFs, the red circle refers to the upregulated DEIRGs, and the green circle refers to the down-regulated DEIRGs. The red line is the positive correlation between TFs and DEIRGs. The green line is the negative correlation between TFs and DEIRGs.

KEGG pathway analysis of DEIRGs in LUSC identified six significant pathways, including antigen processing and presentation, cytokine–cytokine receptor interaction, natural killer cell-mediated cytotoxicity, graft-*versus*-host disease, viral protein interaction with cytokine and cytokine receptor, and chemokine signaling pathway (**Figure 6A** and **Supplementary Table S10**). GO analysis of DEIRGs in LUSC identified 81 significant BPs, 5 CCs, and 12 MFs (**Figure 6B** and **Supplementary Table S12**). PPI network showed some of the high combined scores (combined score > 0.9) between those DEIRGs in LUSC, for example, CALCA and IAPP, ELANE and BPI, ELANE and RNASE3, KLRC1 and KIR2DL1, KLRC1 and KIR2DL3, CCL16 and CCL1, RNASE3 and BPI, KLRC1 and KLRC4, CALCA and GPHA2, KIR2DL3 and KIR2DL1, CCL16 and MCHR2, MCHR2 and CCL1, and GPHA2 and IAPP (**Figure 6C** and **Supplementary Table S14**).

### Potential TF-DEIRG Regulatory Networks in LUAD and LUSC

To explore the potential upstream regulatory mechanism of DEIRGs, RNA-sequencing data of TFs were selected from TCGA database based on Cistrome DB in LUAD and LUSC (**Supplementary Tables S15**, **S16**), respectively. Subsequently, correlation analysis was carried out between TFs and DEIRGs, and only significant correlations with p < 0.05 and correlation coefficient > 0.4 were selected as results, which were shown for LUAD (**Figure 5D** and **Supplementary Table S17**) and LUSC (**Figure 6D** and **Supplementary Table S18**). In LUAD, 26 TFs were identified as potential upstream regulatory mechanisms of DEIRGs, including ASCL1, BACH2, BRCA1, CEBPA, CENPA, CIITA, E2F1, ETV1, EZH2, FOXM1, FOXP3, GATA6, IKZF1, IRF4, MYBL2, MYH11, NCAPG, PPARG, RBP2, RXRG, SCML2, SOX17, SPIB, STAT4, TAL1, and TCF21. In LUSC, 29 TFs were identified as potential upstream regulatory mechanisms of DEIRGs, including BATF, CIITA, EOMES, EPAS1, ESR1, ETS1, FLI1, FOXA2, FOXP3, GATA6, HNF1B, HNF4A, IKZF1, IRF1, IRF4, MEF2C, MITF, NFE2, NR5A2, RBP2, RUNX1T1, SOX17, SPIB, STAT4, TAL1, TCF21, TCF7, and VDR.

### Construction of Prognostic Model for LUAD

**Figure 7:**
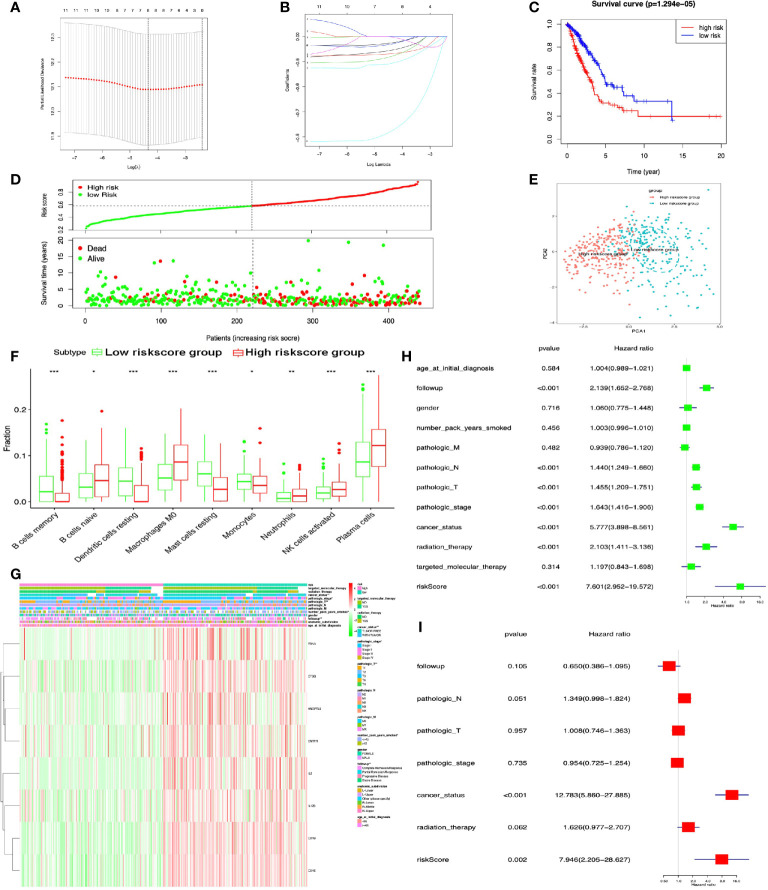
Lasso regression identified the prognostic model in LUAD. **(A, B)** Lasso regression complexity was controlled by lambda with glmnet R package. **(C)** OS analysis between high- and low-risk score groups. **(D)** Risk plot between high- and low-risk score groups. **(E)** Principal component analysis (PCA) for risk scores revealed two completely disjoint populations, suggesting that there existed extensive differences in the landscape of risk scores between high- and low-risk score samples. Blue means low-risk score samples; red means high-risk score samples. **(F)** Boxplot showed the ratio differences of nine immune cells between high- and low-risk score groups in LUAD, and Wilcoxon rank sum was used for the significance test. **(G)** The heatmap of clinical correlation between high- and low-risk score groups in LUAD. **(H)** The univariate Cox regression analysis of risk factors in LUAD. **(I)** The multivariate Cox regression analysis of risk factors in LUAD. *p < 0.05, **p < 0.01, and ***p <0.001.

DEIRGs in LUAD were used for COX regression analysis to select OS-related DEIRGs for lasso regression analysis, and 11 DEIRGs were significantly related to OS, including ANGPTL5, ANGPTL7, CCL17, CD1A, CD1B, CD1E, CNTFR, CTSG, EDN3, IL12B, and IL2 (**Supplementary Table S19**). Among these OS-related DEIRGs, the eight-immune-related gene-signature model (ANGPTL5, CD1B, CD1E, CNTFR, CTSG, EDN3, IL12B, and IL2) was established with lasso regression to improve the predictive accuracy for OS in LUAD, when log (lambda) was between −4 and −5 (**Figures 7A, B** **)**. Based on this eight-immune-related gene-signature model, LUAD tissue samples were divided into high- and low-risk score groups according to the mean value of risk scores (**Supplementary Table S20**). Additionally, OS showed statistical significance between high- and low-risk score groups (**Figures 7C, D** **)**. All LUAD samples were well-divided into two groups (high- and low-risk groups) according to risk scores based on PCA verification (**Figure 7E**). The distribution of immune cells was significantly different between high- and low-risk score groups in LUAD, including B cells naive, B cells memory, dendritic cells resting, macrophages M0, monocytes, mast cells resting, neutrophils, plasma cells, and NK cells activated (**Figure 7F**). The heatmap showed that the high-risk group had a significant association with clinical features, including follow-up, number of pack years smoked, pathological T, pathological stage, and cancer status (**Figure 7G** and **Supplementary Table S21**). The eight-immune-related gene signature was consistent with single-factor Cox regression analysis of gene. The univariate Cox regression analysis revealed that follow-up, pathological N, pathological T, pathological stage, cancer status, radiation therapy, and risk score were significantly correlated with OS (**Figure 7H**). The multivariate Cox regression analysis revealed that cancer status [hazard ratio (HR) = 12.783, 95%CI (5.860–27.855), p < 0.001] and risk score [HR = 7.946, 95%CI (2.205–28.627), p = 0.002] possibly acted as an independent risk factor in LUAD (**Figure 7I**).

### Construction of Prognostic Model for LUSC

**Figure 8:**
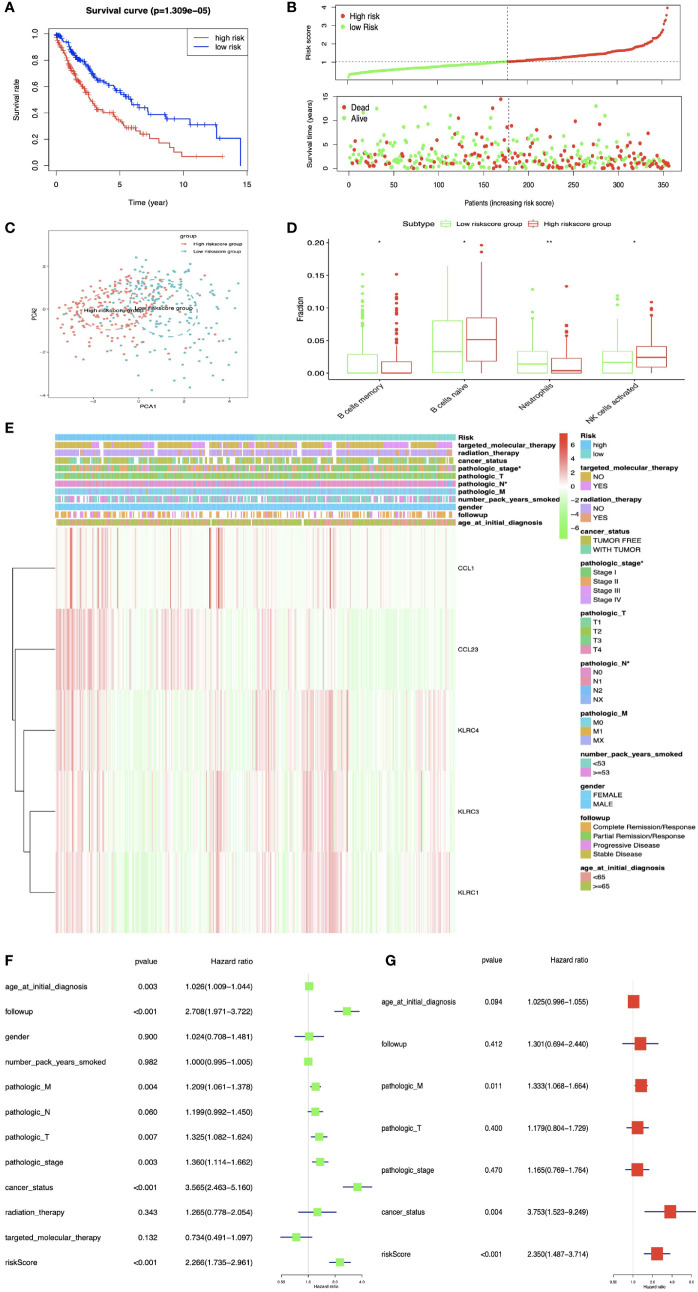
The multiple linear regression identified the prognostic model in LUSC. **(A)** OS analysis between high- and low-risk score groups. **(B)** Risk plot between high- and low-risk score groups. **(C)** Principal component analysis (PCA) for the risk scores. Blue means low-risk score samples; red means high-risk score samples. **(D)** Boxplot showed the ratio differences of four immune cells between high- and low-risk score groups in LUSC, and Wilcoxon rank sum was used for the significance test. **(E)** The heatmap of clinical correlation between high- and low-risk score groups in LUAD. **(F)** The univariate Cox regression analysis of risk factors in LUSC. **(G)** The multivariate Cox regression analysis of risk factors in LUSC. *p < 0.05, **p < 0.01.

DEIRGs in LUSC were used for multivariate COX regression analysis to select OS-related DEIRGs to construct prognostic model, and the five-immune-related gene-signature model (CCL1, KLRC3, KLRC4, CCL23, and KLRC1) was established with multivariate COX regression to improve the predictive accuracy for OS in LUSC (**Supplementary Table S22**). Based on this five-immune-related gene-signature model, all LUSC samples were divided into high- and low-risk score groups according to the mean value of risk scores (**Supplementary Table S23**). Additionally, OS showed statistical significance between high- and low-risk score groups (**Figures 8A, B** **)**. All LUSC samples were divided into high- and low-risk groups according to risk score based on PCA verification (**Figure 8C**). The distribution of immune cells was significantly different between high- and low-risk groups, including naive B cells, B cells memory, and NK cells activated (**Figure 8D**). The heatmap showed that high-risk group had a significant association with clinical features, including pathological N and pathological stage (**Figure 8E** and **Supplementary Table S24**). The five-immune-related gene-signature was consistent with single-factor Cox regression analysis of gene. The univariate Cox regression analysis revealed that follow-up, pathological M, pathological T, pathological stage, cancer status, and risk score were significantly correlated with OS (**Figure 8F**). The multivariate Cox regression analysis revealed that cancer status [HR = 3.753 95%CI (1.523–9.249), p = 0.004], pathological M [HR = 1.333, 95%CI (1.068–1.664), p = 0.011], and risk score [HR = 2.350, 95%CI (1.487–3.714), p < 0.001] possibly acted as an independent risk factor in LUSC (**Figure 8G**).

### Correlations Between DEIRG Expression in Prognostic Model and Clinical Data in LUAD and LUSC

**Figure 9:**
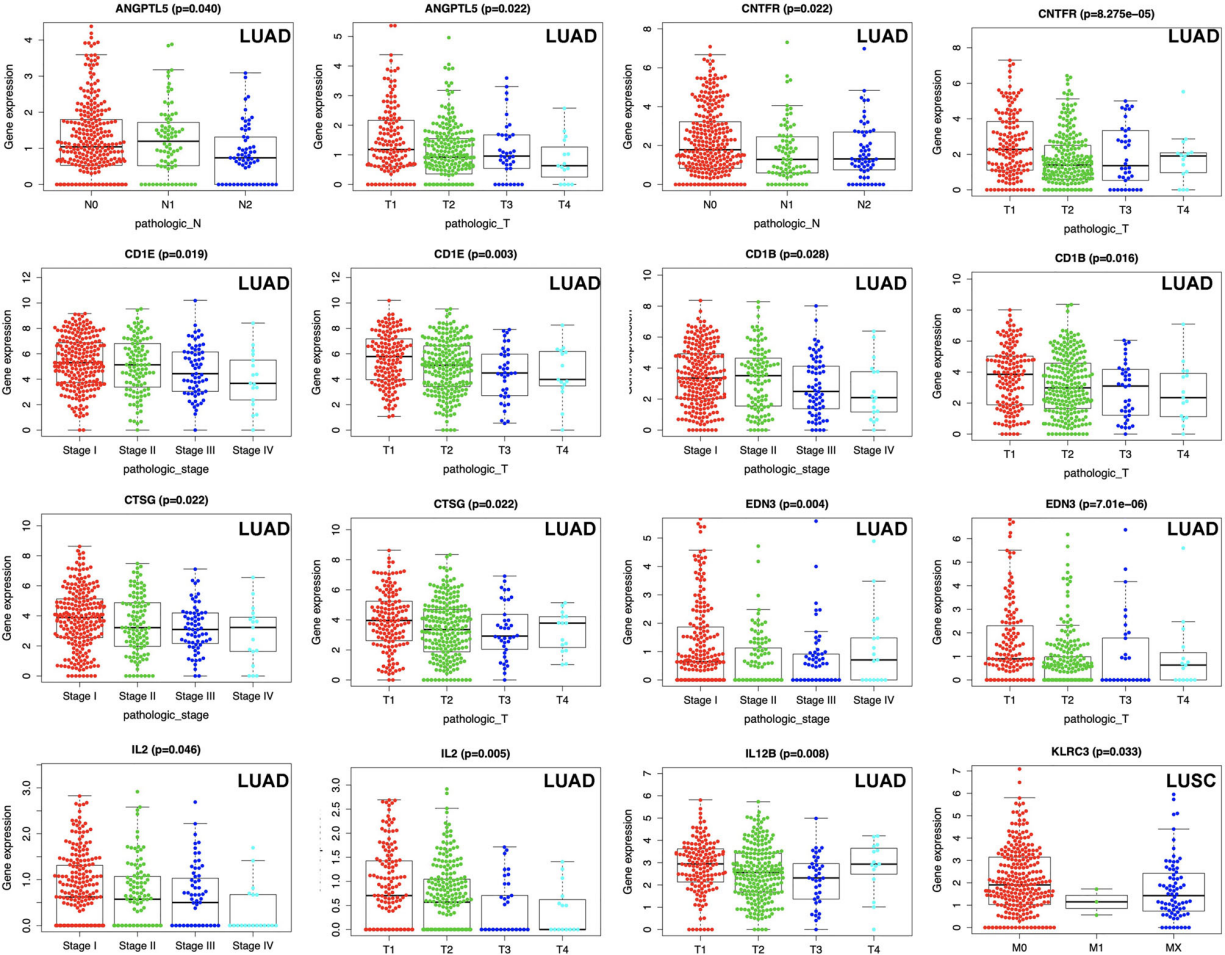
Correlations between DEIRG expressions in prognostic model and clinical data in LUAD and LUSC.

The TNM system (including pathological T, pathological N, pathological M, and pathological stage) was the most commonly used cancer staging evaluation method for cancer diagnosis. In LUAD, each DEIRG expression from prognostic model was significantly correlated with TNM system, including ANGPTL5 and CNTFR with pathological N and pathological T; CD1B, CD1E, CTSG, EDN3, and IL2 with pathological stage and pathological T; and IL12B with pathological T (**Figure 9**). In LUSC, only KLRC3 expression from prognostic model was significantly correlated with pathological M in TNM system (**Figure 9**).

## Discussion

With in-depth study of CSC theory, it was found that CSCs had (i) the obvious characteristics such as self-renewal and unlimited proliferation; (ii) pathological behaviors to participate in radio-resistance, metastasis, relapse, and drug resistance; and (iii) extensive clinical application in diagnosis and treatment, in lung cancers (22, 23). One study separated CSCs with single-cell assay of human LUAD cancer cells (A549), with CD44+/CD24−/phenotype characteristics, special stemness-related gene expression, and Hoechst 33342 dye efflux assay, and the secondary protein structure of CSCs changed when compared to non-stem cancer cells (24). It clearly demonstrated that CSCs might be prognostic biomarkers in cancer therapy (24). Multidrug resistance and epithelial–mesenchymal transition (EMT) were closely associated with the existence of CSCs, which offered promise in developing reliable CSC markers of NSCLC-associated multidrug resistance and EMT. Some studies began to focus on specific stemness-related pathways such as Wnt/β-catenin pathway and revealed the mechanism of malignant phenotype (25). A CSC-targeted therapy with good biocompatibility and targeting capability was developed to disrupt the growth and survival of CSCs, which might provide an effective approach to prevent the relapse caused by CSCs (26). A study on CSC might provide valuable clues to solve the poor prognosis of lung cancer. Some lung cancer CSCs might be retained, but not eliminated, under the traditional therapies. The interaction between TME and CSCs might provide new clues to treat CSCs in a cancer (27). Especially in TME, the relationship between lung CSCs and tumor-infiltrating lymphocytes was very marginally considered. High ALDH expression was a key biomarker for CSCs. Immunohistochemistry revealed statistically positive correlations between ALDH+ and CD8+ and between ALDH+ and CD3+ cells populations. CSCs existed in different lymphocyte distribution, which might contribute to precision medicine management of different kinds of lymphocytes (28). CSCs also showed obvious properties to shape TME and help themselves to escape recognition from the immune system and become immunosuppressive (29). In turn, tumor-infiltrating immune cells also affected the status of tumor cells. For example, inflammatory factors [TGF-β combined with tumor necrosis factor alpha (TNF-α)] could induce tumor cells to turn CSCs with high expression of CD133, CD44, Bmi1, and Oct4 (30). CD4+ CD25+ Tregs were well-known to reduce antitumor immunity, and Tregs increased side population of mouse breast cancer cells, promoted sphere formation, and upregulated stemness-related genes. It revealed a certain interaction between Tregs and CSCs, which suggested that targeting the crosstalk between Tregs and CSCs was a promising strategy (31). Thus, better understanding of individualized immune signature and tumor immunology of CSCs might be one of the most effective treatments under the background of immunotherapy strategy (32).

This study analyzed the associations between cancer stemness and TME in lung cancer, which identified DEIRGs between high- and low-mRNAsi subtypes in LUAD and LUSC, respectively. The literature reported that some DEIRGs played an important role in stemness processes. Interleukin-27 (IL27) encoded one of subunits of a heterodimeric cytokine complex (33). The IL27–IL12B complex drove rapid expansion of naive CD4(+) T cells and trigger interferon gamma (IFNG) production of naive CD4(+) T cells. IL-27 downregulated stemness-related genes, such as SONIC HEDGEHOG in LUAD cells, and NOTCH1, SOX2, KLF4, Nestin SNAI2/SLUG, OCT4A, SNAI1/SNAIL, and ZEB1 in LUSC cells (33). Additionally, clinical data showed that even immunocompromised or advanced NSCLC patients could benefit from the treatment of IL-27 based on its ability to downregulate stemness-related genes (33). Bone morphogenetic protein 7 (BMP7) encoded a member of TGF-beta family of proteins. The function of BMP7 was to activate a dormant state in cancer cells by p38 MAPK signaling and p21 expression to reduce stemness (34).

C–C motif chemokine ligand 1 (CCL1) belonged to chemokine CC family and was secreted by activated T cells and displayed chemotactic activity for monocytes. CD4+ CD25+ Tregs could reduce antitumor immunity and had a crosstalk with CSCs. The activated NF-κB-CCL1 signaling could recruit CD4+ CD25+ Tregs by reducing the binding of H3K27Me3 on promoter regions of p65 and Ccl1, and recruited CD4+ CD25+ Tregs increased the expressions of stemness-related genes (31). Heparin-binding growth factor 8 (FGF8) encoded by this gene was a member of the fibroblast growth factor (FGF) family. FGF family members played crucial roles in mitogenic and cell survival activities. FGF8 was also reported to dramatically enhance stemness (35). Activated macrophages (M2) secreted chemokines, such as chemokine ligand 17 (CCL17), whose expression was significantly associated with clinical pathological characteristics of hepatocellular carcinoma and with poorer overall survival rates. The stemness could be examined by flow cytometry, sphere formation, and Western blot. When cancer cells treated with CCL17, stemness-related markers were highly expressed, and EMT process was enhanced (36). The cytokine colony-stimulating factor 2 (CSF2) controlled production, differentiation, and function of granulocytes and macrophages. Myeloid-derived suppressor cells (MDSCs) were one kind of immune cells leading to tumor immune escape, which was verified with cell colony formation, tumor sphere formation, and CSC biomarkers (NANOG and c-MYC). MDSCs could induce CSCs and promote tumor immune evasion in different kinds of cancers through CSF2/p-STAT3 signaling pathway. When CSF2 was deleted in tumor cells, cell stemness could be markedly reversed through downregulating CSF2 expression (37). Further studies on the interactions between immune system and CSCs and targeting on the key point among them might be a promising strategy in cancer therapy.

This study found that DEIRGs between high- and low-mRNAsi subtypes in LUAD and LUSC were enriched in some crucial signaling pathways. Some enriched signaling pathways were reported to play an important role in stemness processes. For example, Janus kinases/signal transducer and activator of transcription (JAK/STAT) signaling pathway took part in cellular processes such as proliferation, cell death, cell division, immunity, and tumor formation, and this pathway could help transfer communication information from extracellular chemical signals to cell nucleus to activate gene transcription process (38). A previous study on microRNA-related signaling pathways contributing to stemness features of CSCs found that JAK/STAT was one of the various targeted signaling pathways that contributed to dysfunctions of stemness-related microRNAs (39). This finding prompted one to further study whether DEIRGs between high- and low-mRNAsi subtypes enriched in JAK/STAT pathway (CNTFR, CRLF2, CSF2, IL12B, IL2, and IL22RA2 in LUAD) affect stemness in lung cancer. The chemokine family depended on seven-transmembrane-spanning G-protein-coupled receptors to generate activity. The diversity of chemokine–chemokine receptor interaction made the function of chemokines become more complicated and plastic. Chemokines and their receptors did not only mediate leukocyte infiltration into TME but also multiple hallmark processes of a cancer. However, chemokines and their receptors were not fully understood yet (40). A study found chemokines to be implicated in contributing to tumor heterogeneity through maintenance or promotion of stem-like phenotype. The CXCR4 (chemokine)/CXCL12 (chemokine receptor) interaction helped to promote tumor-initiating cells in lung carcinomas, which was associated with resistance to chemotherapy (41). DEIRGs between high- and low-mRNAsi subtypes enriched in cytokine–cytokine receptor interaction pathway (BMP7, CCL17, CCR9, CNTFR, CRLF2, CSF2, IL12B, and IL2 in LUAD, and CCL16, CCL1, IL2, CCNA2, IL26, CCL23, IL27, and DOLKL in LUSC) could provide potential and novel cytokines and cytokine receptors for CSC study. Antigen processing and presentation was a vital immune process to trigger T-cell immune response. The recognition of fragmented antigens in tumor cells by immune cells depended on major histocompatibility complex (MHC) and related receptors on immune cell surfaces (42). Studies showed that immunogenicity of CSCs was lower than non-stemness cancer cells, and CSCs were good at concealing their specific antigens to escape the immune effect of immune cells (43). Therefore, it was necessary to recognize and increase the specific antigen on the surface of tumor stem cells to increase immune. Renin–angiotensin system (RAS) was commonly known to regulate systemic vascular resistance, body fluid homeostasis, and blood pressure. However, RAS was also involved in maintenance and differentiation of CSCs, which showed that identification of RAS components in CSCs could provide a novel way for therapeutic targeting with RAS modulators in common clinical use (44). DEIRGs between high- and low-mRNAsi subtypes enriched in RAS-related pathways (CMA1 and CTSG in LUAD) were consistent with previous studies. Recently, a great improvement has been achieved from immune checkpoint therapy; for example, immune checkpoint therapy can kill CSCs (45). PD-L1 was one of definite checkpoints for tumor cells to escape from immune surveillance because PD-L1 binds PD-1 receptor to block T cell function. A study found that PD-L1 was highly expressed in CD133+CD44+ colorectal CSCs, and the altered PD-L1 could upregulate the expressions of stemness-related genes to increase CSC self-renewal ability to form tumor spheres. Thus, anti-PD-L1 monoclonal antibodies might crucially contribute to kill CSCs. DEIRGs between high- and low-mRNAsi subtypes enriched in antigen processing and presentation pathway (KLRC4, KIR2DL1, KLRC3, KLRC1, KIR2DS4, and KIR2DL3 in LUSC) could offer much more possible mechanisms of immunological process of CSCs. Further study on the interaction between immune-related pathways and CSCs would make potential mechanism of immunotherapy more clearer in cancer therapy.

The interaction between CSCs and immune cells and alteration of tumor-infiltrating immune cells around CSCs were observed in various cancers (27). This study found that the distribution of immune cells between high- and low-mRNAsi subtypes were significantly different, including B cells memory, dendritic cells resting, macrophages M0, mast cells resting, monocytes, plasma cells, T cells CD4 memory resting in LUAD, and macrophages M1, plasma cells, T cells CD4 memory resting, T cells CD4 memory activated, and T cells CD8 in LUSC. Some of these immune cells were reported to play an important role in stemness processes. For example, cellular colocalization of cathepsins B and D on CSCs and cathepsin G on mast cells suggested that the altered distribution of mast cells was observed in TME CSCs (46). The efficiency of dendritic cell (DC)-based treatment targeting CSCs was tested in breast cancer treatment. After DCs pulsed with breast CSC total RNA, total lymphocytes were cocultured with CSC total RNA-pulsed DCs and populations of lymphocytes and were analyzed with flow cytometry. The results showed that cocultured productions contained cytotoxic CD8 T lymphocytes, CD4 T lymphocytes, NK, and NKT cells (47). Tumor-associated macrophages, one population of key immune cells in TME, were closely associated with the progression of NSCLC. In terms of molecular mechanisms, macrophage-derived IL-10 activated JAK1/STAT1/NF-κB/Notch1 signaling to enhance properties of stemness in NSCLC. In turn, when IL-10/JAK1 signaling was blocked, CSC-related genes might be reversed (48). Monocytes, as the largest type of leukocyte, differentiated into dendritic cells and macrophages. Even though they belonged to innate immune system, monocytes also involved in the process of adaptive immunity. Tumor-associated monocytes created a CSC niche through juxtacrine secretion pathway to enhance CSC activities of carcinoma cells (49). Hepatocarcinoma cells HepG2 were cocultured with mouse splenic B cells (MSBCs). Those HepG2 cells were verified to obtain stem-cell-like characteristics, such as high tumorigenic capacity, self-renewal, extensive proliferation, overexpression of CSC-related genes and proteins, drug resistance, and highly activated Notch and SHH signaling pathways (50). CD8+ T-cells-mediated antitumor immunity was one of developed strategies to target CSCs. One previous study isolated CSCs from human lung cancer cell line H460 with special marker ALDEFLUOR, and CSC lysate-pulsed dendritic cells was used to stimulate CD8+ T cells as a treatment strategy. The results indicated that ALDH-high-CD8+T cells might directly target against ALDH-high CSCs to mediate antitumor immunity (51). This study found that the distribution of immune cells was significantly different between high- and low-risk score groups in LUAD and LUSC, respectively. Most of these results were coincident with the distribution of immune cells between high- and low-mRNAsi subtypes. Moreover, immune cells might recognize, attack, and eliminate cancer cells, and they might also have the ability to induce a small population of cancer cells to acquire stem cell-like characteristics. Our results revealed that CSC-cell-based tumor-infiltrating immune cell immunotherapy might be clinically useful.

TFs were involved in regulating “turn on and off” genes by binding to a specific DNA sequence. Groups of TFs had diverse effects throughout the life of the cell and organism, such as cell migration, division, growth, death, and organization during embryonic development (52). To explore the underlying correlation between DEIRGs and TFs, this study screened TF-DEIRG regulatory networks. A total of 26 TFs in LUAD and 29 TFs in LUSC were identified as potential upstream regulatory mechanisms of DEIRGs. Some of these identified TFs in LUAD and LUSC were closely associated with stemness in lung cancer. For example, forkhead box M1 (FoxM1) was involved in cell proliferation and regulated the expression of cell cycle genes (cyclin B1 and cyclin D1). A study found that the upregulation of TF FoxM1 mediated the acquisition of cancer stem-like cell characteristics in NSSLC H460 cells with analysis of stemness markers (CD133, ALDH1, and CD44) (53). Enhancer of zeste 2 polycomb repressive complex 2 subunit (EZH2) was one of the members of polycomb-group (PcG) family, which played roles in maintaining the transcriptional repressive state of related genes during cell division (54). EZH2 altered stem-like phenotypes and progression of lung cancer *via* regulating the malignant gene modifier (histone methyltransferase) (55). Nuclear receptor NR5A2 was a regulator of stemness of pluripotent stem cells and embryonic stem cells, which was also reported to promote CSC properties and tumorigenesis in NSSLC by regulating Nanog (56). Hypoxia-inducible factor-2 alpha (HIF-2α) was involved in the induction of genes regulated by oxygen, which was induced by decreased oxygen levels. Hypoxia was considered to be one of the most important factors in TME. Most malignant tumors produced a hypoxic microenvironment that was conducive to their own development, which suppressed immune response (57). Lung cancer stem-like cells [CD133(+)] A549 were divided from cultured cells with serum-free culture conditions by fluorescence-activated cell sorting. Tissue sections from 50 NSSLC cases were used to estimate the correlation between HIF-2α levels and lung cancer stem-like cells [CD133(+)] with immunohistochemical analysis. HIF-2α levels were significantly higher in CD133(+) cells compared to CD133(−) cells, and HIF-2α caused radioresistance of CSCs (58). For this study, the construction of TF-DEIRG regulatory networks provided some molecular mechanisms of CSC processes in lung cancer.

Most CSCs are dormant and have strong drug resistance and insensitivity to radiotherapy. The traditional chemotherapy drugs and radiotherapy might not kill them effectively, which results in tumor metastasis and recurrence. Due to the development and application of immunotherapeutic drugs such as targeting PD-1 and PD-L1, immunotherapy has become one of the main treatment of lung cancer. As an effective therapeutic method, cellular immune intervention can target tumor-specific antigen and/or tumor-associated antigen and specifically remove tumor cells or CSCs. This study analyzed three levels of associations between mRNAsi and immunity, including (i) the associations of immune-related scores and mRNAsi, (ii) the associations of immune cells and mRNAsi, and (iii) differently expressed immune-related genes (DEIRGs) between high mRNAsi and low mRNAsi groups. Those provided potential immune molecular markers and mechanisms for immunotherapy in CSCs. In summary, this study focused on the alterations of immune-related genes and immune cells through the acquisition of stemness characteristics based on mRNAsi in LUAD and LUSC. Usually, it takes two to tango when ones explore the interaction between stemness and immunity. Two different prognostic models in LUAD and LUSC could be useful to further investigate their clinical application in lung cancers.

## Data Availability Statement

The datasets presented in this study can be found in online repositories. The names of the repository/repositories and accession number(s) can be found in the article/**Supplementary Material**.

## Ethics Statement

Written informed consent was not obtained from the individual(s) for the publication of any potentially identifiable images or data included in this article. All original data were extracted from public database.

## Author Contributions

NL designed the project, analyzed data, prepared figures and tables, and wrote the manuscript. YL and PZ participated in the data analysis. XZ conceived the concept, supervised results, critically revised/wrote manuscript, and was responsible for its financial supports and the corresponding works. All authors contributed to the article and approved the submitted version.

## Funding

This work was supported by the Shandong First Medical University Talent Introduction Funds (to XZ), the Hunan Provincial Hundred Talent Plan (to XZ), the Shandong Provincial Natural Science Foundation (ZR202103020356 to XZ), the National Natural Science Foundation of China (82172866), and the Academic Promotion Program of Shandong First Medical University (2019ZL002).

## Conflict of Interest

The authors declare that the research was conducted in the absence of any commercial or financial relationships that could be construed as a potential conflict of interest.

## Publisher’s Note

All claims expressed in this article are solely those of the authors and do not necessarily represent those of their affiliated organizations, or those of the publisher, the editors and the reviewers. Any product that may be evaluated in this article, or claim that may be made by its manufacturer, is not guaranteed or endorsed by the publisher.
